# Gastrointestinal duplication masquerading as intussusception in an adult

**DOI:** 10.1093/jscr/rjab226

**Published:** 2022-01-20

**Authors:** Patricia Nguyen, Robert Azar, Greg Starley, Elana Scharff

## Abstract

Gastrointestinal duplication is a rare congenital condition that involves tissues anywhere along the alimentary tract. It may also be referred to as enteric duplication or alimentary tract duplication. It typically presents in children and is hardly found in adults because the presentation varies and the diagnosis is often incidental. Surgical resection is generally indicated to prevent future complications. We present a case of duplicated small bowel discovered during surgery in a young female with the initial diagnosis of small bowel intussusception on CT.

## INTRODUCTION

Gastrointestinal duplication is a rare anomaly with an estimated incidence of 1/4500 where part of the intestines is essentially replicated and attached along the alimentary tract [1]. Other sources claim that the incidence may be as low as 1/10 000 live births [2]. Most cases reported up to date have been pediatric, most commonly among the newborn population. They can be identified via ultrasound during prenatal care, and over 80% of cases are discovered by the age of 2 years old [1]. Known duplications may be cystic or tubular in nature, and by definition, they must contain both smooth muscle and gastrointestinal mucosa [3]. The duplicated structure is often non-functioning and benign, but it can share the same blood supply as the native, confluent tract. While it can present early as abdominal masses in children, it can cause nonspecific symptoms such as pain, anorexia, nausea and vomiting among adults [4]. The symptomatology depends on the site and size of the duplication, but most cases are located in the small intestine, particularly at the ileum [2]. Because symptoms are nonspecific, GI duplication can often be misdiagnosed as other conditions such as Crohn’s Disease or mesenteric cysts [5].

## CASE REPORT

**Figure 1:**
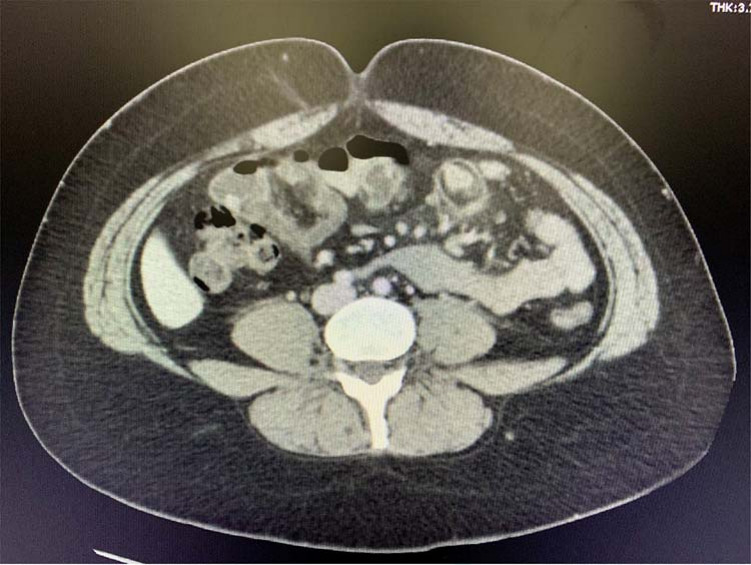
CT consistent with small bowel intussusception and inflammatory changes at the mesentery.

A 29-year-old female presented to the emergency room after several days of worsening epigastric and RUQ abdominal pain, nausea and loss of appetite. On examination, her abdomen had mild tenderness to palpation in the epigastric region and no tenderness in the RLQ. Murphy’s sign was negative. Vital signs were all within normal limits, and the remainder of her physical exam was unremarkable. White blood cell count was elevated, and CT was consistent with small bowel intussusception and inflammatory changes at the mesentery (Fig. 1). The patient was admitted to the hospital for one night, where she was observed. Her pain and nausea did not improve. The decision was made to proceed with surgery.

**Figure 2:**
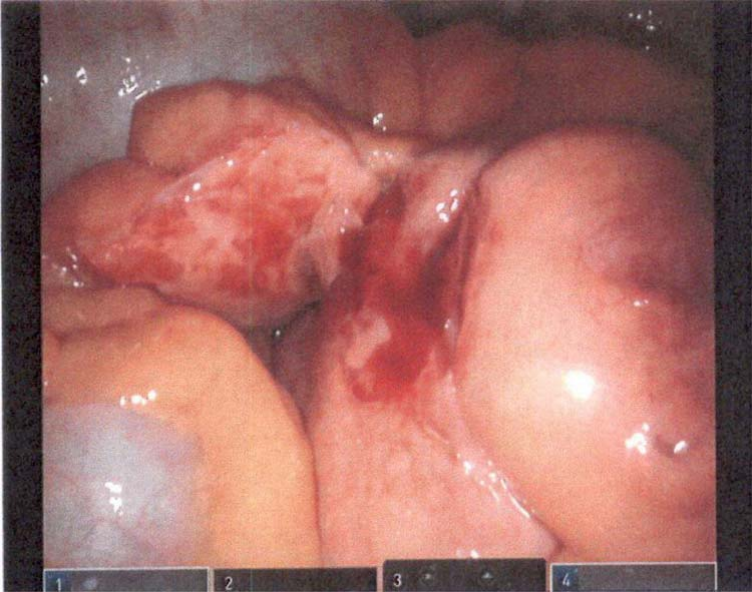
Mesentery appears to be noticeably thickened.

The procedure began with a robotic-assisted laparoscopic approach. The precise anatomy was difficult to ascertain laparoscopically. It was not consistent with intussusception. The mesentery was notably thickened as well (Fig. 2). In order to identify the anatomy safely, the surgery was converted to a laparotomy.

**Figure 3:**
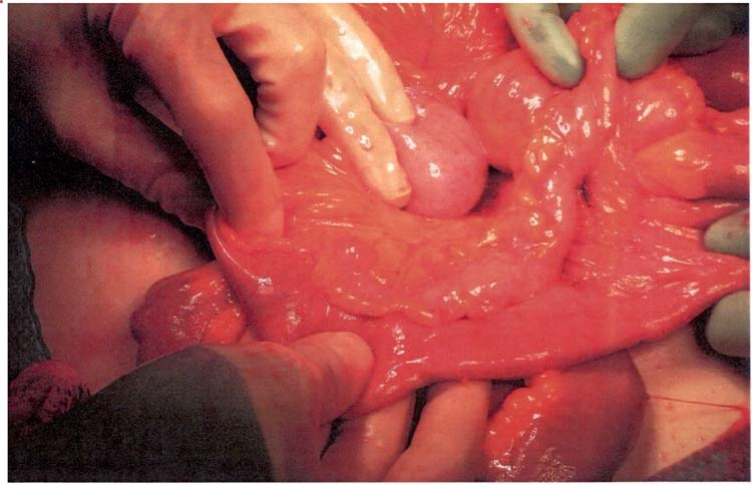
Medial tract presumed to be a duplicated portion of small bowel.

When following the small bowel proximally from the terminal ileum back toward the stomach, a Meckel’s diverticulum was encountered. Just proximal to this Meckel’s diverticulum, there appeared to be two ends of small bowel that diverged. The medial loop of bowel became smaller and more thickened as it approached the proximal mesenteric root where it ended in a blind pouch. This medial tract appeared to be a duplicated portion of small bowel (Fig. 3). A small abscess pocket and several thickened lymph nodes were also discovered near the mesenteric root.

The proximal end of the medial loop was dissected from the mesenteric root, tied off with 0 silk suture and transected distally. The segment of bowel containing the Meckel’s diverticulum and adjacent duplication was resected. A side-to-side anastomosis was performed.

Patient progressed well post-operatively and was discharged on hospital Day 5.

Pathology revealed benign small bowel consistent with duplication and Meckel’s diverticulum.

## DISCUSSION

While relatively rare among adults, gastrointestinal duplication may be encountered at surgical exploration. This patient had no significant past-medical history and did not have any previous surgeries. CT imaging of the abdomen suggested the presence of a small bowel intussusception in the left hemiabdomen. Comparison of the CT with intra-operative findings reveals how the duplication was misinterpreted as intussusception.

Since intussusception within adults is typically transient, this patient’s dense regional inflammatory changes and prominent lymph nodes in the central mesentery suggested an alternate etiology for the CT findings. Like most cases of gastrointestinal duplication, the diagnosis was determined intra-operatively.

Current recommendations for managing gastrointestinal duplication involve surgical intervention, specifically resection and primary anastomosis. This approach aims to preserve common blood supply to the native bowel and other peripheral organs [4]. Additionally, it may prevent complications including distension, hemodynamic instability, ulceration, intussusception, volvulus, bowel obstruction and malignant degeneration [2]. Segmental resection is often curative and leads to favorable prognosis. Nevertheless, specific guidelines are not well-established due to the sparse number of cases reported. Ileal duplications make up 60% of all known alimentary tract duplications, and only 27 cases of ileal duplication have been in the current literature [2]. For our patient, the anatomy allowed for segmental resection of the duplication, Meckel’s and adjacent small bowel. This preserved the blood supply and prevented future complications. Although intestinal duplication in an adult is rare, general surgeons should be familiar with its management.
